# Targeting Glutamate Neurotoxicity through Dietary Manipulation: Potential Treatment for Migraine

**DOI:** 10.3390/nu15183952

**Published:** 2023-09-12

**Authors:** Fahimeh Martami, Kathleen F. Holton

**Affiliations:** 1 Department of Health Studies, American University, Washington, DC 20016, USA; fmartami@american.edu; 2 Department of Neuroscience, American University, Washington, DC 20016, USA; 3 Center for Neuroscience and Behavior, American University, Washington, DC 20016, USA

**Keywords:** excitotoxicity, neuroinflammation, oxidative stress, migraine, nutrients, diet

## Abstract

Glutamate, the main excitatory neurotransmitter in the central nervous system, is implicated in both the initiation of migraine as well as central sensitization, which increases the frequency of migraine attacks. Excessive levels of glutamate can lead to excitotoxicity in the nervous system which can disrupt normal neurotransmission and contribute to neuronal injury or death. Glutamate-mediated excitotoxicity also leads to neuroinflammation, oxidative stress, blood-brain barrier permeability, and cerebral vasodilation, all of which are associated with migraine pathophysiology. Experimental evidence has shown the protective effects of several nutrients against excitotoxicity. The current review focuses on the mechanisms behind glutamate’s involvement in migraines as well as a discussion on how specific nutrients are able to work towards restoring glutamate homeostasis. Understanding glutamate’s role in migraine is of vital importance for understanding why migraine is commonly comorbid with widespread pain conditions and for informing future research directions.

## 1. Introduction

Migraine is a common neurological disorder with prevalence rates ranging from 9–16% worldwide [1]. This disorder primarily occurs during the most productive years of adulthood, from age 20 to 50 years [2]. According to the latest report from the Global Burden of Disease study, migraine is the primary cause of disability among people less than 50 years of age [3]. In 2016, the annual cost of healthcare utilization and lost productivity associated with migraine in the US was estimated at $36 billion [4]. Migraine is associated with a wide spectrum of comorbidities including gastrointestinal, psychiatric, cardiac, and cerebrovascular disorders, which can increase the physiological burden [5]. Despite the profound impact of migraine, it is still underdiagnosed and undertreated [6,7].

The pathophysiological mechanism underlying migraine is not completely understood. However, activation and sensitization of meningeal nociceptors in the trigeminovascular (TG) system are widely accepted as a key pathway in the initiation of a migraine attack [8]. The TG system is comprised of sensory neurons that originate from the trigeminal ganglion that innervate cerebral blood vessels including the dura mater, the outermost layer of the meninges [9].

Evidence supports the role of glutamate neurotransmission in both the activation and perpetuation of migraine [10,11]. Peripheral release of glutamate is involved in the generation of migraine pain through N-methyl-D-aspartate (NMDA) receptors found in the meningeal afferents of the trigeminal nerve [12]. High glutamatergic activity also leads to increased cerebral excitability and resultant cortical spreading depression (CSD) that can cause nociception in the dura mater [10,13]. Interestingly, in addition to the direct effect of glutamate in the activation of trigeminal nociceptors and the contribution to CSD development, it is also contributing to pain sensitization as well. Previous reports have indicated that the production and release of the vasodilatory neuropeptides calcitonin gene-related peptide (CGRP) and substance P (SP) can be induced by increased glutamatergic neurotransmission [14,15]. Perivascular release of these neuropeptides can eventually lead to a phenomenon called “neurogenic inflammation” which is believed to be an underlying element leading to sensitization of trigeminal meningeal nociceptors [16]. Central sensitization has been observed in migraine and can lead to contact allodynia (i.e., pain from a stimulus that does not ordinarily cause pain) [17]. Central sensitization is an augmentation of membrane excitability through upregulation of glutamatergic neurotransmission, which contributes to pain hypersensitivity in many pain conditions [18]. The NMDA glutamate receptor is pivotal for both the initiation and maintenance of central sensitization, and thus, in reverse, is also thought to be the key in stopping this process [19]. A high concentration of glutamate in the synaptic cleft can lead to excitotoxicity, which is the over-excitation of neurons which leads to apoptosis, or cell death [20]. Excitotoxicity causes oxidative stress and inflammation in the central nervous system (CNS). The reinforcing properties between excitotoxicity, oxidative stress, and neuroinflammation (the “neurotoxic triad”) have been implicated in neurologic disorders including chronic pain and migraine [21]. Thus, disorders such as migraine may benefit from interventions targeting glutamate specifically. 

Therefore, in the current review, we aim to combine existing knowledge about the role of glutamate in migraine pathogenesis along with information on nutrients that are protective against glutamate excitotoxicity, and then end with a proposed dietary treatment for migraine management.

## 2. Glutamatergic Neurotransmission

Glutamate is the main mediator of excitatory neurotransmission in the brain. It has two types of receptors, ionotropic and metabotropic [22]. Ionotropic glutamate receptors (iGluRs) are ligand-gated channels subcategorized into NMDA, the α-amino-3-hydroxy-5-methyl-4-isoxazole propionate (AMPA), and the kainic acid (KA) receptor, which all have fast excitatory effects. Metabotropic receptors (mGluRs) are G-protein-coupled receptor channels. Eight metabotropic receptors have been characterized (mGluR1-8) and fall into three groups (group I-III) based on their similarity regarding second messenger systems and pharmacology, with group I being associated with slow excitation, and groups II and III being more associated with slow inhibition [22]. 

Glutamate receptors are found throughout the body; therefore, dysregulation of the glutamatergic system can impose a broad range of effects [22]. In the nervous system, glutamate is implicated in crucial aspects of normal brain function including synaptogenesis, learning, cognition, and memory [23,24]. As mentioned above, the iGluRs are involved in fast synaptic responses to glutamate, while the mGluRs play a role in slow neuromodulatory signaling [25]. Although these receptors have local and functional variability, glutamate excitotoxicity targets both families of receptors [25]. Excitotoxicity results from the excessive synaptic release of glutamate and the consequent accumulation of high concentrations of free calcium (Ca^2+^) in the cytosol [26] which is mediated by NMDA receptors [26]. However, AMPA and KA receptors can also contribute to Ca^2+^ overload through their partial permeability to Ca^2+^ [26]. The mGluRs function in two ways, by directly increasing cytosolic Ca^2+^ levels via the facilitation of Ca^2+^ release from the intracellular stores, and indirectly by promoting NMDA receptor migration to the cell membrane [27]. Excitotoxicity is one of the leading causes of neuronal damage and death, and this process has been implicated in a variety of neurological diseases including schizophrenia, Alzheimer’s, Parkinson’s, multiple sclerosis (MS), epilepsy, chronic pain, and migraine [28,29,30,31,32,33].

## 3. Physiological and Anatomical Evidence Related to the Role of Glutamate in Migraine

**Figure 1:**
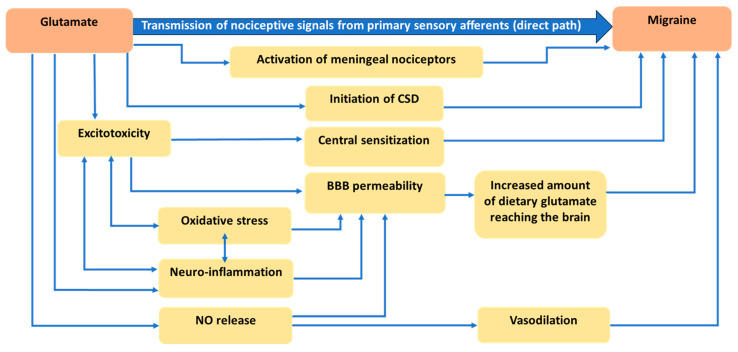
Mechanisms explaining the role of glutamate in migraine pathogenesis.

Figure 1 illustrates the mechanisms involving glutamate in the pathogenesis of migraine (which is reviewed in detail in this section).

### 3.1. Role of Glutamate in Nociception

The trigeminal system (in addition to C1 and C2 fibers) comprises all of the nociceptive neurotransmission within the head [34]. C1 and C2 fibers refer to the first and second cervical spinal nerves that, along with second-order neurons of the trigeminal nucleus caudalis, comprise the trigeminocervical complex [8]. Nociceptive signals from the meninges and cervical roots are sent to higher-order brain regions including the brainstem, hypothalamus, basal ganglia, and thalamus [8]. Projection of trigeminovascular thalamic neurons to different areas of the cortex contributes to pain perception as well as migraine-associated symptoms [8]. Glutamate has a well-known role in the transmission of nociceptive signals from primary sensory afferents to second-order neurons in the brainstem [35]. 

Interestingly, evidence from preclinical studies has shown that all types of glutamate receptors are characterized and found in the trigeminal system [36,37,38,39]. In line with these findings, other studies have also indicated that the administration of glutamate leads to hyperalgesia [40,41] while inhibition of glutamate blocks its nociceptive effects [42]. An interesting in vivo experiment explored the role of peripherally released glutamate in the generation of migraine pain by using trigeminal neurons from animal models [12]. They found that glutamate and aspartate (another amino acid with a similar structure that also functions as a neurotransmitter) can activate NMDA receptors on peripheral sensory trigeminal ganglion neurons in meningeal nerve terminals and that they can induce excitation of meningeal afferents implicated in the generation of migraine pain [12]. Thus, glutamate can lead to trigeminal nociception in two ways: (1) via signal transmission from primary meningeal nerves to higher brain regions, which results in cortical excitability, and (2) via activation of NMDA receptors in tissues located outside of the blood-brain barrier (BBB), which consequently leads to trigeminal nociception [12].

### 3.2. Role of Glutamate in Cortical Spreading Depression

Glutamate is also proposed as a key player in the initiation of CSD, which is an expanding wave of depolarization (activation of neurons) followed by a wave of hyperpolarization (inactivity of neurons while concentration gradients re-set) across the cortex [43,44]. Preclinical evidence supports the role of CSD in stimulating trigeminal neurons [45]. Local release of glutamate by neurons is assumed to trigger CSD [46]. CSD is recognized as the biological reason for migraine aura which has been further confirmed with the observation of CSD waves in migraine aura patients [47]. The latest version of the International Classification of Headache Disorders (ICHD) (Third edition) defines migraine aura as an “early symptom of an attack, believed to be the manifestations of focal cerebral dysfunction, typically lasting 20–30 min and precedes the headache” [48]. Visual aura is, by far, the most prevalent type of aura [49]. 

### 3.3. Role of Glutamate in Central Sensitization

The intensity and duration of headache attacks are attributed to the development of central sensitization [50]. Central sensitization is defined as abnormal amplification in central nociceptive processing because of increases in membrane excitability as well as reduced inhibition [50]. Central sensitization is observed in both chronic and episodic migraine [51]. Excitotoxicity is considered a major player in the onset and continuation of central sensitization [15]. This is thought to occur via the upregulation of NMDA and AMPA receptors on the primary afferent neurons, with a subsequent reduction in the threshold for neuronal activation, which contributes to the onset of central sensitization [52]. Higher levels of glutamate in plasma have been observed in both chronic and episodic migraine patients as compared to healthy controls, with no significant difference between chronic and episodic migraineurs [53]. Cutaneous allodynia, which is highly prevalent in migraine patients, is a clinical manifestation of central sensitization [54]. Interestingly, cutaneous allodynia has been associated with response to preventive treatment, with severe occurrence being associated with decreased response to treatment [55,56,57]. This evidence supports the important role of central sensitization in migraine pathophysiology.

### 3.4. Role of Glutamate in Disruption of the Blood-Brain Barrier (BBB)

The integrity of the BBB guarantees a unique environment for the CNS by controlling what substances can enter and leave the CNS [58]. Therefore, disruption of the BBB allows the influx of potentially toxic substances into the brain. BBB permeability can occur via the overactivation of NMDA receptors which causes excitotoxicity [59], but other events such as head trauma [60], infection [61], neurotoxic exposures [62], high stress [63], neuroinflammation [64], and oxidative stress [65] can also lead to permeability of the BBB. The contribution of neuroinflammation and oxidative stress, which are tightly tied to excitotoxicity, will be described in more detail below.

Increased glutamatergic neurotransmission in migraine leads to the sustained secretion of vasoactive substances including CGRP and SP [66]. These neuropeptides contribute to vasodilation, plasma protein extravasation, mast cell activation, and the release of proinflammatory cytokines, resulting in a phenomenon called neurogenic inflammation [16], which, as mentioned above, can lead to BBB permeability [67]. 

In neurons, the influx of excess Ca^2+^ into the cell following the activation of glutamate receptors eventually leads to the production of free radicals (atoms missing an electron) which are called reactive oxygen species (ROS) and reactive nitrogen species (RNS) [68]. Further support for this idea is provided by electron paramagnetic resonance spectroscopy showing that NMDA receptor activation results in the production of superoxide radicals [69]. Typically, ROS and RNS are counteracted by antioxidants or antioxidant enzyme systems within the cell, which have the ability to donate an electron to re-balance the free radicals. If the amount of free radicals outstrips the antioxidant defense system of the cell, oxidative stress occurs [70]. These free radicals can start a cascade of events including mitochondrial impairment, and damage to lipids, proteins, and DNA, leading to mutagenesis, and ultimately cell death [71]. Additionally, as mentioned above, oxidative stress can lead to BBB permeability [65]. 

Increased permeability of the BBB in migraine patients could result in the entry of blood-borne toxins, as well as increased amounts of dietary glutamate and aspartate, into the brain, which could elicit excitotoxicity. It is noteworthy that excitotoxicity, neuroinflammation, and oxidative stress have the ability to perpetuate one another, allowing this “neurotoxic triad” to be maintained over time. Thus, neurogenic inflammation and oxidative stress can also be involved in migraine initiation/sensitization through potentiating excitotoxicity.

### 3.5. Role of Glutamate in Nitric Oxide Release and Vasodilation 

A link between glutamate and nitric oxide (NO) was initially proposed after the finding that glutamate or NMDA treatment causes the release of NO and cyclic guanosine monophosphate (cGMP) in cerebellar cultures [72]. Additionally, NMDA receptor activation results in a rise in cGMP levels in the brain, with nitric oxide synthase (NOS) inhibitors and NO scavengers preventing this rise in cGMP levels [72]. This suggests that NO has signaling functions downstream of NMDA receptor activation. In neurons, Ca^2+^ entry, as a result of the activation of NMDA receptors, induces NOS, which is physically coupled to NMDA receptors [73]. Glutamate can also activate NMDA receptors in the endothelial cells of capillaries, causing subsequent induction of NOS, and release of NO, which causes vasodilation [74]. The contribution of cerebral and meningeal arterial vasodilation in migraine initiation has been suspected for many decades [75,76]. Interestingly, NO can negatively affect the BBB when it combines with a superoxide radical to form peroxynitrite, a potent free radical that leads to oxidative stress and excitotoxicity [77,78,79]. 

## 4. Glutamate Concentration in Migraineurs

Increased levels of glutamate in plasma [80,81,82], cerebrospinal fluid (CSF) [14,82,83], and platelets [84,85,86] have been detected in migraine patients. This elevated level of glutamate was observed during attacks as well as during interictal periods [82,83,87], for those with and without aura [82,88,89]. Furthermore, a meta-analysis on excitatory neuro-metabolite levels across pain conditions, using data pooled from magnetic resonance spectroscopy studies, revealed a significant increase in glutamate levels in the brains of migraine patients, compared with controls [90]. This evidence could reflect cortical neuronal hyperexcitability and points to the dysfunction of glutamatergic signaling in migraine pathogenesis. In a study by Ferrari et al., prophylactic medications lowered the frequency of attacks and glutamate levels compared to baseline; however, migraine sufferers still had higher serum levels of glutamate compared to healthy controls [89]. Another case-control study among migraine patients without aura, using proton magnetic resonance spectroscopy, showed an increased level of the glutamate/glutamine ratio between attacks in both the primary occipital cortex and thalamus [91]. Table 1 represents the glutamate concentration in adult migraine patients, as compared to healthy controls, in various tissues. Significantly higher glutamate concentrations have been reported in the plasma, platelet, CSF, and brain of migraine patients, as compared to healthy controls.

## 5. Dietary Components Affecting Glutamate Neurotoxicity and Migraine

**Figure 2:**
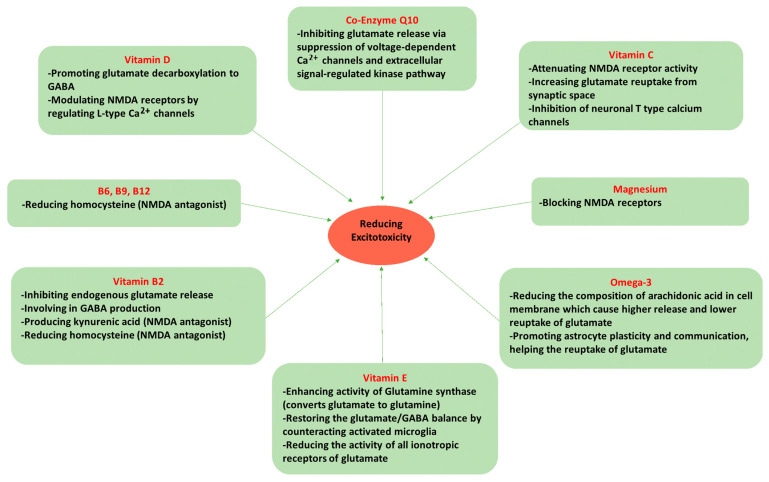
Nutrients mechanism in protecting against excitotoxicity.

Dietary factors may be one of the most important modifiable lifestyle components for treating migraines. There are specific micronutrients that protect against excitotoxicity caused by excess glutamate. These same micronutrients have also shown promising efficacy in migraine reduction in clinical settings. These nutrients are reviewed below (Figure 2 illustrates the protective mechanisms for each nutrient against excitotoxicity). 

While outside the scope of this review, it should also be quickly noted that dietary factors can also affect the microbiome and that these important gut bacteria may also be influential in migraine. For a comprehensive review of what is known about the gut-brain axis in migraines, please refer to [97].

### 5.1. Omega-3 Fatty Acids

Omega-3 fatty acids are long-chain, polyunsaturated fatty acids that contribute to normal brain development and function [98]. Docosahexaenoic acid (DHA), a very long chain omega-3 fatty acid, has been identified as an important component of the lipid membrane of the CNS and an abundant phospholipid in the gray matter of the cerebral cortex [98]. Besides their structural function, omega-3s are a precursor for signaling molecules, as well as playing a role in neurotransmission and gene expression [99].

Both in vivo and in vitro evidence have shown beneficial effects of omega-3 derivations on nociception [100,101,102]. The essential omega-3 fatty acid, alpha-linolenic acid, showed a neuroprotective effect against glutamate-mediated excitotoxicity, a critical cause of neuronal injury in animal studies, including epilepsy [103], ischemia [104], stroke [105] and spinal cord injury [106]. One of the earliest studies providing evidence regarding the neuroprotective potential of omega-3 fatty acids was derived from an animal study investigating the effect of an omega-3-supplemented diet on neuronal damage, as compared to a control diet (using olive oil). The neuronal injuries were induced by middle cerebral artery occlusion and infusion of an NMDA receptor agonist, by the researchers. Rats supplemented with omega-3s had significantly reduced damage in both focal ischemia and excitotoxicity [107]. The underlying mechanism of this effect could be attributed to the change in membrane fatty acid composition. Arachidonic acid (an omega-6 fatty acid) has been reported to be associated with increased excitotoxicity by inducing a prolonged inhibition of glutamate reuptake into glial cells [108] and also increased release of glutamate into the synaptic cleft [109]. Therefore, substitution of omega-3 fatty acids for omega-6 could offer beneficial effects on excitotoxic brain damage. Additionally, eicosapentaenoic acid (EPA) and DHA (long-chain omega-3s) also showed promising benefits for protecting against monosodium glutamate (MSG) neurotoxicity in the hippocampus of prepubertal rats [110]. This neuroprotective effect of omega-3s could be attributed to their role in enhancing the plasticity, communication, and function of astrocytes [111]. Astrocytes are the major regulators of glutamate homeostasis and prevent excitotoxicity by taking glutamate up out of the synaptic cleft. This effect is supported by the finding that a lack of omega-3s can aggravate the negative impact of aging on astroglial morphology and activity [112]. In summary, omega-3 fatty acids may be effective in reducing excitotoxicity, making this an important class of nutrients for neurological protection.

Epidemiological research has shown an inverse association between dietary intake of omega-3s and the prevalence and characteristics of headache disorders including migraine [113,114]. Human clinical studies that investigated the potential effects of omega-3s on migraine suggest that omega-3 supplementation might improve migraine-related outcomes [115,116,117]. A meta-analysis indicated that omega-3s significantly reduced migraine duration; however, no significant change in terms of frequency or intensity was detected [118]. 

### 5.2. Magnesium (Mg^2+^)

Magnesium is an important intracellular mineral that plays vital roles in a wide range of metabolic reactions [119]. Magnesium is also critical for normal CNS function. It is involved with nerve transmission, the release of neurotransmitters, and protection against excitotoxicity [120]. Low levels of magnesium have been reported in many neurological disorders including Alzheimer’s disease [121], traumatic brain injury [122], stroke [123], epilepsy [124], Parkinson’s [125], psychiatric disorders [126], and migraine [127]. Low brain magnesium was also detected during a migraine attack using magnetic resonance spectroscopy in migraine patients [128]. 

There are several mechanisms underlying the anti-nociceptive effect of magnesium especially related to glutamate-mediated excitotoxicity. Magnesium blocks NMDA glutamate receptors, thereby protecting against excitotoxicity, and since NMDA receptor antagonists suppress trigeminal nociceptive transmission, this mineral could be a potential modulator of trigeminovascular nociception [34]. In a rat model of trigeminovascular activation, blocking NMDA receptors with either magnesium or memantine (an antagonist of NMDAR) inhibited nociceptive activation of the trigeminocervical complex [129]. In support of this effect on the NMDA receptor, a reduction in damage was observed in magnesium-treated mice who had induced excitotoxicity by ibotenate, a glutamate receptor agonist [130]. Moreover, in an animal model of cerebral ischemia, the extracellular level of glutamate in the cortex was reduced following magnesium administration [131]. In experimental models, magnesium also had an inhibitory effect on CSD [132,133] and deficiency in this mineral increases the sensitivity of NMDA receptors to glutamate-mediated CSD [134]. 

The effectiveness of magnesium has been extensively evaluated for migraine prevention. The results of a meta-analysis of randomized clinical trials indicated that oral magnesium significantly alleviated the frequency and severity of migraine, and intravenous magnesium was effective in relieving acute migraine attacks [135]. However, another meta-analysis investigating the effects of intravenous magnesium failed to show a beneficial effect in terms of pain relief [136]. 

### 5.3. Vitamin D

Vitamin D is a steroid hormone that is best known for its role in Ca^2+^ and phosphorus homeostasis and osteogenesis [137]. Notably, the beneficial effects of vitamin D extend well beyond mineral absorption and bone health. It is considered a neurosteroid because of its crucial role in neuronal integrity and brain development [138]. Vitamin D deficiency has been linked to neurological disorders [139]. Vitamin D receptors are broadly found in different parts of the brain including the cortex, hypothalamus, thalamus, hippocampus, and substantia nigra, supporting the potential role of vitamin D in different neurological conditions [140]. 

In vitro evidence has demonstrated the protective effects of vitamin D against glutamate excitotoxicity [141], which may be partially mediated by vitamin D’s role in gene transcription, affecting the production of key enzymes in the nervous system. Vitamin D deficiency can reduce glutamate decarboxylase levels in the brain. Glutamate decarboxylase is the enzyme that catalyzes the decarboxylation of glutamate to convert it into γ-aminobutyric acid (GABA), the main inhibitory neurotransmitter in the nervous system [142]. Thus, vitamin D can help prevent excitotoxicity indirectly by upregulating the production of the enzyme that increases the conversion of excitatory glutamate into inhibitory GABA. Notably, vitamin D may also reduce excitotoxicity via modulation of NMDA receptors by regulating Ca^2+^ influx through L-type voltage-sensitive Ca^2+^ channels [143]. 

Epidemiological studies evaluating serum levels of vitamin D in migraine patients have reported conflicting results, with some case-control studies showing no differences between migraine patients and healthy controls [144,145], and others observing significant differences [146,147]. However, a meta-analysis in 2020 summarizing the results from 8 observational studies reported overall significantly lower serum levels of 25(OH)D (the main circulating form of vitamin D) in migraine patients, as compared to healthy controls [148]. Additionally, the concentration of vitamin D in the blood has also been associated with migraine characteristics, as migraine patients with vitamin D deficiency are more likely to suffer frequent and severe attacks than migraine patients with adequate levels of vitamin D [146,149]. Vitamin D administration was found to be effective in alleviating migraine-related outcomes in a meta-analysis of five randomized controlled trials [150]. 

### 5.4. Vitamin C

Vitamin C, or ascorbic acid, is a water-soluble vitamin known mostly for its unique antioxidant properties [151]. Vitamin C has a critical role in antioxidant defense as well as many non-antioxidant activities in the CNS [151]. 

Ascorbic acid exerts a neuroprotective effect against excitotoxicity through attenuating NMDA receptor activity [152] and increasing glutamate reuptake from the synaptic cleft [153]. Vitamin C has also been shown to reduce oxidative stress induced by monosodium glutamate (MSG). In an experimental study on albino rats, vitamin C supplementation protected against degenerative changes in neurons and astrocytes in the cerebellar cortex induced by MSG [154]. Vitamin C also selectively inhibits T-type calcium channels in peripheral and central neurons, which are involved in the control of neuronal excitability [155]. Additionally, vitamin C neutralizes ROS, effectively addressing the oxidative stress caused by excitotoxicity. Therefore, it appears that vitamin C may possess multiple neuroprotective properties.

Despite the limited number of studies concerning the role of vitamin C in migraines, the evidence presented above supports the potential of vitamin C in fighting excitotoxicity, thereby preventing migraines. To date, the only randomized controlled trial related to this research area is a small pilot study that administered N-acetylcysteine, vitamin E, and vitamin C in migraine patients. They showed that this antioxidant combination significantly reduced the frequency, intensity, and duration of attacks, as well as the number of acute medications being used, as compared to the controlled group [156]. Clearly, more research is needed on vitamin C’s efficacy in migraine reduction.

### 5.5. Vitamin E

Vitamin E is a generic term for compounds called tocopherols and tocotrienols. Alpha-tocopherol is the main form (with the highest biological activity) found in human and animal tissue [157]. Vitamin E has been extensively studied for its antioxidant properties, as the dominant lipid-soluble, chain-breaking antioxidant in the body, which supports membrane integrity by preventing lipid peroxidation [157]. The brain has very high amounts of polyunsaturated fatty acids, making vitamin E essential for the antioxidant protection of these lipids [158]. 

In an experimental model of neuropathic pain, vitamin E had an analgesic effect by reducing central sensitization [159]. Vitamin E showed potential for fighting excitotoxicity, reducing glial cell activation, neuronal death, neuroinflammation, and oxidative stress in the hippocampus, in an epilepsy model [160,161]. A possible underlying mechanism is attributed to the regulatory effect of vitamin E on glutamine synthase activity, which is believed to be suppressed by oxidative stress [160,162]. Glutamine synthase converts glutamate to glutamine, a non-excitotoxic amino acid, to allow it to be safely shuttled from astrocytes to neurons before being recycled back to glutamate [162]. Vitamin E can also be involved in glutamate and GABA balance through counteracting microglial activation and the inflammatory cascade [163,164]. The cytokines released from microglia affect neuron excitability by modulating astrocytic glutamate receptors and transporters [165]. Substantial evidence from rodent and human studies indicates that inflammation causes downregulation of glutamate decarboxylase activity, which results in a lower conversion of glutamate into GABA, increasing the likelihood of excitotoxicity occurring [166,167,168,169]. In line with this evidence, it was shown that transgenic mice, which express increased levels of pro-inflammatory cytokines or chemokines, had lower levels of glutamate decarboxylase in the hippocampus and cerebellum [170]. Therefore, the anti-inflammatory effects of vitamin E may protect GABA production and vulnerability to more excitation. Furthermore, in vitro evidence has demonstrated that vitamin E reduces astrocytes’ permeability to Ca^2+^ and Na^+^ ions by inhibiting protein kinases and downregulating glutamate receptor genes [171]. 

Vitamin E, as a potential treatment option for migraine, has only been studied in regard to menstrual migraines [172]. A double-blind, placebo-controlled, crossover clinical trial indicated that vitamin E supplementation for five days during two menstrual cycles was associated with significant improvement in pain severity and functional disability [172]. A probable explanation for vitamin E efficacy as a prophylaxis of menstrual migraine is related to its inhibitory effect on phospholipase A2 and cyclooxygenase enzymes. This leads to inhibition of arachidonic acid release from cell membranes and its conversion to prostaglandin [173]. High levels of prostaglandin have been reported in the endometrium during menstruation and in the serum during the premenstrual phase [174]. The inhibitory effect of vitamin E on phospholipase A2 is of substantial value since there is evidence showing that the enzyme targets other intracellular membranes including the mitochondrial membrane as well [175]. Mitochondrial membrane damage is associated with high ROS production, oxidative stress, and ultimately cell death [176]. It is worth noting that antioxidants work together to maintain themselves in an active state, so despite their unique functions in redox balance, they can also be indirectly involved in each other’s activity as well. The benefits described in the aforementioned study looking at the combined effects of vitamin E, vitamin C, and N-acetylcysteine, may have partially been due to these interactive effects of combining antioxidants [156]. 

### 5.6. Riboflavin (Vitamin B2)

Riboflavin, also known as vitamin B2, is involved in various metabolic pathways through two coenzyme forms including flavin adenine dinucleotide (FAD) and flavin mononucleotide (FMN) [177]. In addition to riboflavin’s critical role in energy metabolism, it also has antioxidant function and plays a pivotal role in the metabolism of vitamin B6 (converting dietary pyridoxine into its active form pyridoxal L-phosphate), as well as having roles in DNA repair, and apoptosis [177]. Therefore, deficiency or any disturbance in riboflavin metabolism can contribute to broad-spectrum dysfunction including cardiovascular, neuromuscular, immune, and neurological abnormalities [177].

Riboflavin has direct and indirect ameliorating effects on glutamate excitotoxicity which is implicated in migraine pain. The reduction in voltage-gated Ca^2+^ channel activity by riboflavin can inhibit endogenous glutamate release by inhibiting glutamate exocytosis in synaptic clefts [178]. In addition, experimental studies demonstrated the neuroprotective effects of riboflavin and pyridoxal phosphate (PLP) on excitotoxicity [179]. As mentioned earlier, riboflavin is involved in the formation of PLP, which is required for the production of many neurotransmitters in the CNS [180]. Very importantly, the conversion of glutamate to GABA, the major inhibitory neurotransmitter in the nervous system, by glutamic acid decarboxylase, necessitates PLP as a cofactor [181]. Therefore, it is not surprising that deficiency in riboflavin and consequent reduction in PLP formation contribute to the elevation of glutamate and reduction in GABA levels, thereby resulting in excitotoxicity. These two vitamins are also crucial for the kynurenine pathway, which is considered the major pathway for the catabolism, or breakdown, of tryptophan [182]. Adequacy of riboflavin and PLP has been linked to the production of kynurenic acid which is a protective antagonist of NMDA and all ionotropic glutamate receptors [183]. Deficiency of these cofactors can lead to further metabolism down the pathway causing the production of quinolinic acid, which is an extremely neurotoxic metabolite that increases the risk of excitotoxicity in multiple ways [184,185]. 

Riboflavin is one of the most studied vitamins for migraine prophylaxis. In this regard, clinical studies on adult migraine patients have shown very promising results [186,187,188]. A pooled analysis of 8 randomized controlled clinical trials indicated a significant reduction in terms of migraine days, frequency, pain intensity, and duration of attacks following 400 mg/day riboflavin supplementation for three months [189]. Currently, the American Academy of Neurology (level B evidence) recommends 400 mg per day for adult migraineurs [190], as compared to the current recommended dietary allowance of 1.1–1.6 mg per day. It should be noted, that as a water-soluble vitamin, the excess is just being excreted (as can be seen by fluorescent-colored urine when you take riboflavin), and thus, such high doses are likely not needed for benefiting migraine patients. Although not all available evidence is obtained from high-quality trials, due to riboflavin’s low cost, high tolerability, and effectiveness in migraine alleviation in the majority of research, it could be considered an advantageous vitamin for migraine [191].

### 5.7. Vitamin B6 (Pyridoxine), Folate (Vitamin B9), and Vitamin B12 (Cobalamin) 

Vitamins B6, B9, and B12 (in addition to riboflavin) play a key role in one-carbon metabolism, and their deficiency has been linked to elevated levels of homocysteine (Hcy) [192]. Hcy is another neurotoxic metabolite that has the ability to activate NMDA receptors, and vitamins B6, folate, and B12 can protect against its accumulation [192,193]. 

An experimental model of pain induced by acetic acid demonstrated the antinociceptive effects of B vitamins [194]. However, it seems the effectiveness of these vitamins in migraine prophylaxis could be attributed to their effect on lowering Hcy for the most part. Notably, the role of riboflavin in the production of the active form of pyridoxine makes it indirectly involved in this pathway as well [192]. The presence of high levels of Hcy in the brain might act as a trigger or amplifier in a variety of ways [195]. Hcy has a known neurotoxic effect via direct stimulation of NMDA receptors and consequent excitotoxicity [193]. Previously, it has also been shown that Hcy acts as an antagonist to GABA-A receptors, influencing the migraine pain threshold negatively [196]. Homocysteine also contributes to the breakdown of the extracellular matrix which affects BBB integrity [196]. An increase in brain microvascular permeability was also observed in mice with hyperhomocysteinemia via the activation of matrix metalloproteinases, which lead to vascular remodeling and BBB disruption [197]. 

Migraine, especially migraine with aura, is associated with a risk of ischemic stroke [198], and elevated levels of Hcy in migraineurs have been identified as a potential risk factor for stroke, as reported by epidemiological studies [199,200]. Evidence showing the effectiveness of B6, B9, and B12 vitamins on Hcy level reduction encouraged trials to explore the beneficial effects of Hcy-lowering vitamins. In a double-blind randomized controlled trial by Askari et al., 3 months of supplementation with folic acid plus pyridoxine in migraine patients with aura, led to significant improvement in migraine characteristics compared to placebo [201]; while in the migraine group that received folic acid alone, no significant change was detected in comparison with the placebo group [201]. One clinical trial tested pyridoxine supplementation for migraine patients with aura, and the authors reported a reduction in the severity and duration of attacks, but no effects on the frequency of attacks were noted [202].

### 5.8. Coenzyme Q_10_ (CoQ10)

CoQ_10_ is a fat-soluble compound mostly found in animal proteins, but also in beans, nuts, seeds, and avocado [203]. Our body can synthesize CoQ10, thus its dietary intake is not considered essential. However, evidence has shown that CoQ10 deficiency can occur secondary to several mitochondrial disorders, aging, and in those using statins (for lowering cholesterol) [204,205]. As mentioned before, migraine patients are prone to mitochondrial dysfunction as a result of excitotoxicity-mediated oxidative stress. CoQ10 plays a key role in energy production in mitochondria and also acts as an antioxidant in cell membranes [203]. Interestingly, it is involved in the restoration of the oxidized form of vitamin E, helping to restore vitamin E’s antioxidant function [206]. 

Preclinical evidence supports the protective effect of CoQ_10_ against excitotoxicity. In a mouse model of glaucoma, a diet supplemented with CoQ10 ameliorated glutamate excitotoxicity and oxidative stress compared to an un-supplemented control diet [207]. In another study, the effect of CoQ10 on the endogenous release of glutamate in the cerebral cortex was evaluated [208]. The findings suggested that CoQ10 inhibited glutamate release from cortical synaptosomes in rats via suppression of the presynaptic voltage-dependent Ca^2+^ channels and extracellular signal-regulated kinase pathway. Water-soluble CoQ10 (Ubisol-Q10) has also been shown to reduce glutamate-induced cell death in an in vitro model [209]. Murine hippocampal neuronal cells were exposed to glutamate, 24 h after Ubisol-Q10 treatment. The results indicated that CoQ10 protects the neuronal cells by preserving mitochondrial function and structure. 

The beneficial impact of CoQ10 supplementation on migraine-related outcomes has been tested in several clinical studies [210,211,212,213]. The pooled result of the most recent meta-analysis of 6 studies supports the idea that CoQ10 supplementation can reduce the frequency and duration of migraine attacks but does not reduce severity [214]. 

## 6. Gap between Pathophysiology of Migraine and Interventions: Where Do We Stand Now?

The contribution of glutamate to neuropathological aspects of migraine has led to the development of several glutamate antagonists as migraine prophylactic drugs [215,216,217,218]. However, these drugs have limited utility and a high probability of side effects [219,220]. 

Clinical trials in migraineurs have provided supportive findings for all reviewed nutrients including riboflavin, folate, pyridoxine, cobalamin, vitamin D, C, E, magnesium, and omega-3 fatty acids. These nutrients have shown potential for alleviating excitotoxicity as well. Given the evidence indicating nutrient deficiency among migraineurs [148,200,221], replenishment of these nutrients seems reasonable. However, dietary nutrients are often studied one at a time, which inhibits potential synergism and cooperative effects between nutrients from being observed. Thus, applying a more comprehensive dietary approach may yield greater results. 

Notably, besides being an endogenous source, glutamate is a non-essential amino acid found in the diet [222]. In a normal situation, the amount of dietary glutamate entering the brain is regulated by saturable transporters on the BBB [223]. However, considering the probability of diminished BBB integrity in migraine patients [224,225,226,227,228], it is likely that the amount of dietary glutamate entering the brain is higher than in healthy individuals. This idea is supported by multiple studies that have shown that MSG administration can induce headaches [229,230,231]. Moreover, some dietary components could have a triggering effect on a migraine attack as reported by patients in epidemiological studies [232,233]. The contribution of dietary triggers in migraines was the basis for the development of elimination diet strategies [234]. However, precisely determining random food triggers is challenging, and a diet that is overly restrictive can have a long-term negative effect on nutritional status [235,236]. Until now, no specific diet has been developed for migraine prevention, and several proposed diets have shown varying levels of efficacy [237]. Taken together, both dietary triggers and nutrient intake might be key to therapeutic benefits in migraine, which has not been possible with current interventions.

**Figure 3:**
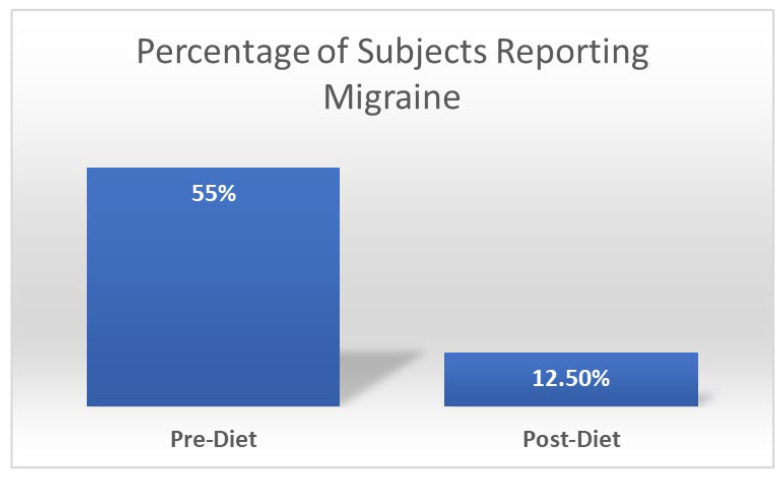
Change in percentage of subjects with Gulf War Illness (*n* = 40) reporting migraine before and after one month on the low glutamate diet. Chi-square significance of *p* = 0.04.

Our team previously administered a diet-based intervention called the “low glutamate diet” in patients with widespread chronic pain disorders [222,238,239]. This diet removes free forms (i.e., not bound to a protein) of glutamate and aspartate (mainly by restricting food additives with excitotoxins, in addition to a few foods that are naturally high in glutamate/aspartate such as soy sauce, fish sauce, and aged cheeses), while also emphasizing the intake of foods high in the micronutrients reviewed above [238]. This diet has shown benefits for widespread chronic pain conditions [222,240], including Gulf War illness (GWI) [238]. Interestingly, all of these studies have demonstrated widespread symptom improvement, including reduced reports of migraines. Figure 3 illustrates the significant reduction in migraine in veterans with Gulf War Illness after one month on the diet. Moreover, most subjects reported going from multiple weekly migraines to no migraines during the diet month. We believe these symptom improvements in patients suffering from widespread chronic pain disorders stem from reductions in central sensitization (from reduced excitotoxicity) and potentially corresponding improvements in the inter-related occurrence of oxidative stress and neuroinflammation. More in-depth research is warranted to further explore whether or not the low glutamate diet may be used as an effective treatment for migraine.

## 7. Conclusions

Glutamate-mediated excitotoxicity is associated with a wide range of neurological disorders including migraine. The proposed mechanisms include the direct effect of excitotoxicity on neuronal injury or death, or its contribution to neuroinflammation, oxidative stress, blood-brain barrier permeability, and cerebral vasodilation, all of which are associated with migraine pathophysiology. Available evidence supports the role of several nutrients in protecting against excitotoxicity including riboflavin, folate, pyridoxine (vitamin B6), cobalamin (vitamin B12), vitamin D, C, E, magnesium, and omega-3 fatty acids. Additional evidence also suggests that supporting endogenous production of CoQ10 with increased dietary intake may also be protective. Interestingly, clinical data support the role of these nutrients in improving migraines as well, providing a strong rationale for designing effective interventions. There is an obvious gap between our understanding of migraines and the dietary strategies which have been administered so far, since dietary nutrients are often studied separately, and no specific diet for migraine has been developed. However, the beneficial effects of the low glutamate diet on widespread chronic pain disorders appear to have overlapping mechanistic effects, and additionally is some preliminary evidence supporting an effect on migraine. Thus, further research on this dietary strategy in migraine is warranted.

## Figures and Tables

**Table 1:** Glutamate concentrations in migraine patients vs. healthy individuals.

	Migraine Patients		Healthy Controls	References
	With Aura	Without Aura		
	Mean (SD)	Mean (SD)	Mean (SD)	
**Plasma**				
Based on aura (nmol/mL)	14 (6)–454 (98)	13 (6)–485 (129)	15 (8)–227 (87)	[53,81,82,85]
Without categorization (nmol/mL)	23 (1)–75 (20)		9 (2)–32 (20)	[80,89,92]
**Platelets**				
(µmol/10^10^ plts)	0.50 (0.22)–0.58 (0.12)	0.43 (0.17)–0.45 (0.16)	0.34 (0.09)–0.45 (0.11)	[84,85]
**Cerebrospinal fluid**				
Based on aura (nmol/mL)	9.3 (1.5)	7.5 (1.8)	4.5 (1.8)	[82]
Without categorization (nmol/mL)	2.18 (0.40)		1.37 (0.30)	[93]
**Brain (MRS)**				
Visual cortex (mmol/l)	6.8 (0.5)	7.0 (0.5)	6.4 (0.8)	[94]
OCC	7.20 (1.45)		6.68 (1.25)	[95]
APC	6.98 (0.85)		6.22 (0.97)	[95]
PCC (mmol)	7.21 (0.96)		7.27 (0.94)	[96]

MRS: Magnetic resonance spectroscopy, OCC: Occipital Cortex, APC: Anterior Paracingulate Cortex, PCC: Posterior cingulate cortex, SD: Standard deviation.

## Data Availability

No new data were created or analyzed in this study.

## Abbreviations

TG: Trigeminovascular, NMDA: N-methyl-D-aspartate, CSD: Cortical spreading depression, CGRP: Calcitonin gene-related peptide, SP: Substance P, CNS: Central nervous system, iGluRs: Ionotropic glutamate receptors, AMPA: α-amino-3-hydroxy-5-methyl-4-isoxazole propionate, KA: Kainic acid, mGluRs: Metabotropic receptors, Ca^2+^: Calcium, MS: Multiple sclerosis, BBB: Blood brain barrier, ICHD: International Classification of Headache Disorders, ROS: Reactive oxygen species, NO: Nitric oxide, cGMP: Cyclic guanosine monophosphate, NOS: Nitric oxide synthase, CSF: Cerebrospinal fluid, DHA: Docosahexaenoic acid, EPA: Eicosapentaenoic acid, MSG: monosodium glutamate, Mg^2+^: magnesium, GABA: γ-Aminobutyric acid, FAD: Flavin adenine dinucleotide, FMN: Flavin mononucleotide, PLP: Pyridoxal phosphate, Hcy: Homocysteine, GWI: Gulf war illness.
